# 24h movement behaviours in university students compared to working age and older adults: wearable-based evidence and cardiometabolic health implications

**DOI:** 10.1186/s12916-026-05192-1

**Published:** 2026-08-29

**Authors:** Ju Lynn Ong, Shuo Qin, Tara H Martin, Xin Yu Chua, Chun Siong Soon, Gizem Yilmaz, Lieng Hsi Ling, Falk Müller-Riemenschneider, Woon-Puay Koh, Michael WL Chee

**Affiliations:** 1 Centre for Sleep and Cognition, Yong Loo Lin School of Medicine, National University of Singapore (NUS), Singapore, Singapore; 2 Department of Cardiology, National University Heart Centre, National University Health System, Singapore, Singapore; 3 Department of Medicine, Yong Loo Lin School of Medicine, National University of Singapore, Singapore, Singapore; 4 Saw Swee Hock School of Public Health, National University of Singapore, Singapore, Singapore; 5 Digital Health Centre, Berlin Institute of Health, Charité– Universitätsmedizin Berlin, Berlin, Germany; 6 Healthy Longevity Translational Research Programme, Yong Loo Lin School of Medicine, National University of Singapore, Singapore, Singapore; 7 Institute for Human Development and Potential, Agency for Science Technology and Research (A*STAR), Singapore, Singapore

**Keywords:** Physical activity, Sedentary behaviour, Sleep, Compositional data analysis, Wearable devices, Obesity, Arterial Stiffness

## Abstract

**Background:**

Physical inactivity, sedentary behaviour and inadequate sleep are established risk factors for non-communicable diseases. Although older adults are traditionally expected to be less active and more sedentary than young adults, contemporary social, occupational and educational environments may have changed this. Here, we characterised 24-hour movement behaviours (physical activity [PA], sedentary behaviour [SB], and sleep) across adulthood using wearable devices and examined their cardiometabolic correlates using a compositional data approach.

**Methods:**

Data were analysed from 1,967 adults across three Singaporean cohorts: university students (*n* = 500; mean[SD] age 19.9[1.3] years), working-age adults (*n* = 437; 41.1[10.0] years), and community-dwelling older adults (*n* = 1,030; 74.0[2.6] years). Each person contributed 4 weeks of Oura Ring recordings on time spent in PA, SB and sleep. BMI, body roundness index (BRI), pulse wave velocity (PWV), and a composite vascular health score (zComposite) were additionally assessed. Associations between reallocations of time across 24 h-movement behaviours and these cardiometabolic outcomes were estimated using compositional data regression models.

**Results:**

University students accumulated the most SB (mean 633.89 min/day) and the lowest PA (320.28 min/day) and sleep (431.75 min/day), whereas older adults accumulated the least SB (500.86 min/day) and greatest PA (420.28 min/day) and sleep (455.62 min/day). Working-age adults showed profiles in-between the students and older adults (SB: 581.28 min/day; PA: 346.55 min/day; sleep: 445.77 min/day). In compositional models, reallocating 30 min per day to PA from other behaviours was associated with lower BMI (-0.13 kg/m²), BRI (-0.04), PWV (-0.05 m/s), and zComposite (-0.01). Reallocating 30 min per day from SB to other behaviours was associated with lower BMI (-0.28 kg/m²) and BRI (-0.07). Reallocating 30 min per day to sleep from other behaviours was related to lower BMI (-0.21 kg/m^2^) and BRI (-0.05).

**Conclusions:**

University students exhibited lower physical activity and higher sedentary time than older adults. Reallocation of time toward greater PA, less SB and longer sleep was associated with lower cardiometabolic risk. These findings challenge conventional expectations about age-related inactivity and highlight young adulthood as a critical, under-recognised target for interventions to promote PA and reduce SB for long-term health.

**Supplementary Information:**

The online version contains supplementary material available at https://doi.org/10.1186/s12916-026-05192-1.

## Background

 Physical inactivity and excessive sedentary behaviour are two leading modifiable risk factors for non-communicable diseases (NCDs) worldwide, contributing substantially to premature mortality and morbidity from cardiovascular disease, type 2 diabetes and obesity [1, 2]. Global estimates suggest that insufficient physical activity accounts for more than 5 million deaths annually [1]. Sedentary behaviour – although related to inactivity, is now recognised as a distinct construct referring to waking behaviours with low energy expenditure, and has been shown to be an independent predictor of cardiometabolic risk beyond physical inactivity [3, 4].

Physical activity has traditionally followed a predictable life-course trajectory: high levels of movement in childhood and early adult life, plateauing during working life and then declining in later years as a result of increased frailty, accumulation of chronic disease, and progressive functional limitations [5–7]. Sedentary behaviours, although generally high in older age [8] tend to peak in working-age adults who have prolonged sitting and commuting demands. This conceptual model, whereby physical activity declines with age while sedentary behaviour peaks in midlife or later, is supported by epidemiological data up until the early 21st century [5–8] and has shaped public health promotion efforts. Whether this life-course trajectory still holds true is of interest.

A third movement behaviour that occupies a significant portion of the 24 h day is sleep. Short sleep has also been consistently linked to increased obesity levels [9–11], insulin resistance [12, 13], and cardiovascular risk [14, 15]. In light of these findings, the American Heart Association added sleep health as the 8th component of its cardiovascular health construct in 2022, acknowledging this as a much broader construct than just the absence of disease [16].

Technological advances in modern society have fostered round-the-clock, global connectivity – be it in study, work or leisure settings. These factors simultaneously decrease time available for physical activity and sleep, while increasing time spent in sedentary behaviours. As such, recent studies have shifted the field away from examining these movement behaviours in isolation, towards recognising these as mutually dependent parts of a finite 24 h day [17]. Using compositional data analysis, early work by Chastin et al. showed that the overall distribution of sleep, sedentary time, light physical activity, and moderate-to-vigorous physical activity (MVPA) was associated with BMI, waist circumference, triglycerides, glucose, and insulin, with the most favourable cardiometabolic profiles generally observed when more time was allocated to MVPA and less to sedentary behaviour [18]. Subsequent studies in adults support these findings, showing that greater relative time in MVPA is associated with lower adiposity, healthier lipid profiles, better cardiorespiratory fitness, and more favourable cardiometabolic risk scores, while sedentary time tends to show adverse associations [19].

However, several gaps remain. Much of the existing evidence is cross-sectional, self-report, derived from Western cohorts, and focused on middle-aged or older adults with richer cardiometabolic phenotyping. Individual studies across multiple countries have shown dramatic declines in physical activity in children and adolescents alongside early and rapid increase in screen time [20, 21], signalling the importance of examining movement behaviours in younger age groups. Studies directly comparing movement behaviour compositions across adult life stages using harmonised objective/wearable-based measures remain uncommon. In addition, relatively few studies have examined both obesity-related and vascular outcomes within the same analytical framework.

Toward these ends, we investigated 24 h movement behaviours across three independent samples in Singapore: university students (18-25y), working-age adults (23-67y), and community-dwelling older adults (67-80y), who were provided with wearable devices. We further analysed how the allocation of physical activity, sedentary time and sleep relate to established cardiometabolic markers of obesity and arterial stiffness [22] within the same, 24-composition analytical framework.

## Methods

### Study design and participants

This analysis draws on data from three independent Singapore-based cohort studies. University students from the National University of Singapore (*n* = 638) took part in the NUS1000 Study [23]; working adults employed by the National University of Singapore (*n* = 575) took part in the NUS1000 Staff Edition Study; while older adults (*n* = 1,205) were drawn from the SG70 cohort [24]. Details of inclusion and exclusion criteria for each study have been described in previous publications [23, 24] and are also provided in Additional File 1: Supplementary Methods.

All participants were provided with Oura Rings, a well-validated wearable health tracker used to assess sleep and activity [25, 26]. The rings were worn on their non-dominant hand, and data across a four-week period (Additional File 1: Supplementary Methods) were averaged for each individual. Questionnaires assessing socio-demographic information and medical history were also obtained during study enrolment, while cardiometabolic health markers (see *Cardiometabolic Measures*) were taken within one month of the Oura Ring data-collection period. All studies were approved by the ethics review board of the National University of Singapore.

### Assessment of 24 h movement behaviours

Key movement behaviours - physical activity (PA), sedentary behaviours (SB) and sleep, were obtained using intraday data available as 5-min metabolic equivalent (MET) epochs based on accelerometry levels from the Oura Ring following proprietary algorithms.^1^ MET epochs were categorized into 5 intensity levels from 4am to 3.59am the next day: 1 = sleep, 2 = SB (1-1.5 METs), 3 = light PA (1.5-3 METs), 4 = moderate PA (3–6 METs), and 5 = vigorous PA (> 6 METs). Duration spent in sleep (category 1), SB (category 2) and PA (category 3–5) was calculated by summing all 5-min epochs corresponding to each category.

Total PA (i.e., the combined duration of light (LPA) and moderate-to-vigorous activity (MVPA)) was modelled as the main exposure variable to: (1) simplify the compositional analysis while preserving interpretability of 24-hour behaviour reallocations, (2) examine the impact of *overall movement* given that we did not have contextual information about the types of activities participants engaged in, and (3) capture total ‘active time’ in a manner that reflects real-world behaviour patterns. Separately, we also conducted sensitivity analyses for LPA and MVPA (see Additional File 1: Supplementary Fig. 1), which showed associations with cardiometabolic markers in the same direction and supports the use of total PA as the main analytic exposure. To further visualize differences in the distribution of activity across 24 h, heatmaps indicating likelihood of daily PA (0 = no PA, 1 = PA) in 5-min bins were plotted and averaged within each group.

Although our primary analyses used 5-min MET categories to classify 24 h movement behaviours, Oura-derived sleep metrics from the main sleep period (i.e. from the longest sleep period in a day) are also presented in Additional File 1: Supplementary Table 1 to facilitate within-sleep period comparisons across age groups. These include: (1) time-in-bed (TIB): representing duration of time spent in bed, (2) total sleep time (TST): representing actual time spent asleep out of the time spent in bed, (3) wake after sleep onset (WASO): denoting time spent awake after the initial sleep onset, (4) sleep fragmentation index (SFI): which quantifies sleep disturbance as the sum of a Movement Index (percentage of movement epochs) and a Fragmentation Index (percentage of sleep epochs lasting ≤ 1 min), and (5) sleep efficiency: 100*TST/TIB.

### Data filtering

**Fig. 1:**
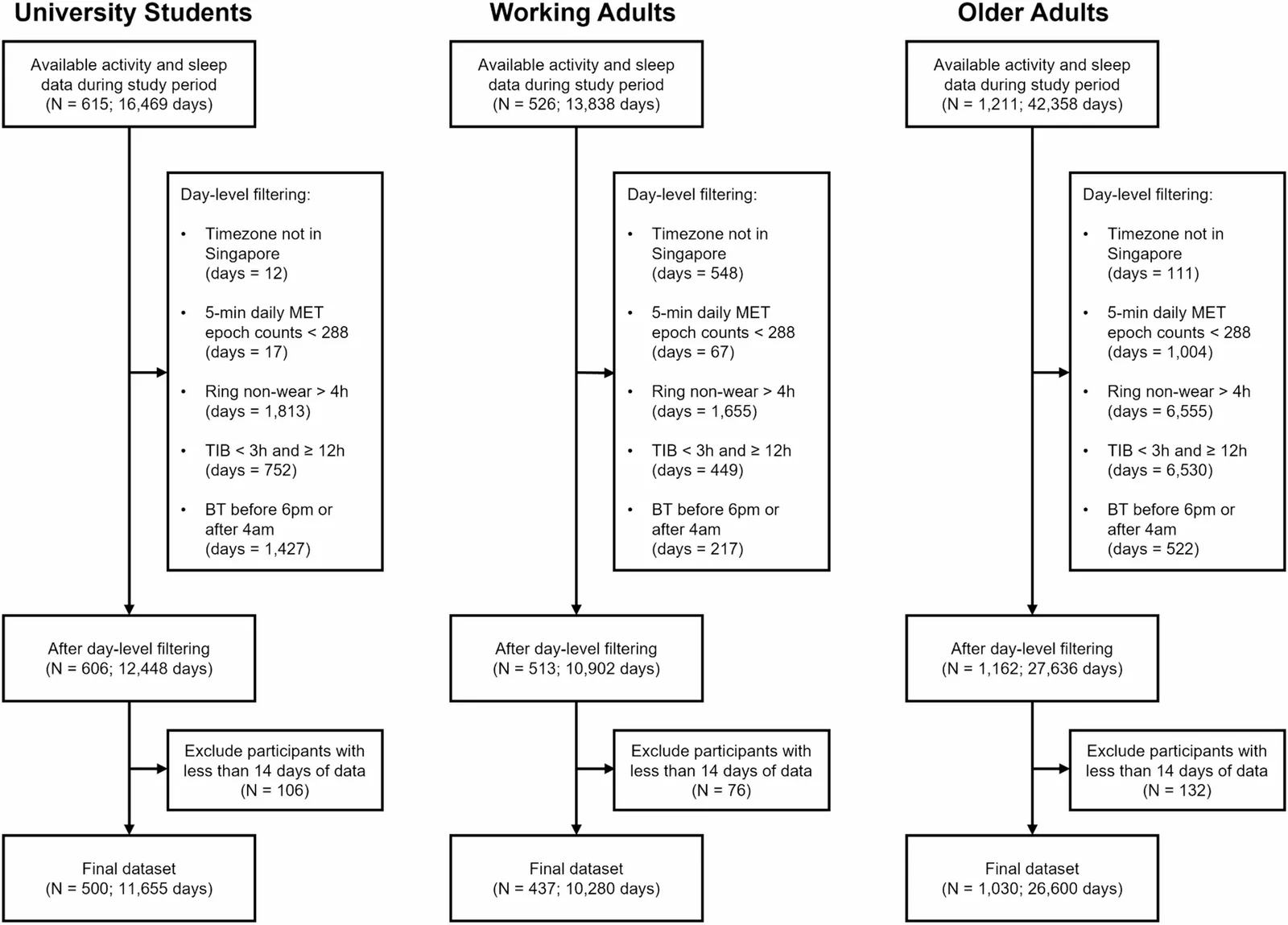
CONSORT diagram illustrating the flow of participants and exclusion criteria across the 3 samples studied (university students, working adults, and older adults). List of abbreviations: MET metabolic equivalent, TIB time-in-bed, BT bedtime

Days were excluded from analysis if: (1) participants were travelling outside of Singapore, (2) 5-min daily MET epoch counts did not span the 24 h day (< 288 epochs) due to battery depletion, (3) ring non-wear was >4 h, (4) main sleep period duration (time in bed) was <3 h or ≥ 12 h which could indicate highly anomalous sleep, or (5) main sleep period bedtime occurred before 6pm or after 4am. Following day-level filtering, participants with fewer than 14 days of valid data remaining were excluded from the analysis (Fig. 1).

### Cardiometabolic measures

Working and older adults underwent assessments for height, weight, and waist circumference, as well as applanation tonometry (SphygmoCor, Cardiex Ltd). Two aspects of cardiometabolic risk were examined in the current study: obesity and arterial stiffness.

To assess obesity, two widely accepted measures were computed: Body Mass Index (BMI) and Body Roundness Index (BRI). Although BMI is widely used, it may underestimate adiposity in older adults due to age-related reductions in lean and bone mass, and may overestimate adiposity in individuals with high muscle mass. BRI has thus been proposed as a more appropriate indicator of obesity [27].

To assess arterial stiffness, carotid-femoral pulse wave velocity (PWV) was determined using a SphygmoCor system (AtCor Medical/CardieX, Sydney, Australia). Additionally, two other indicators of arterial stiffness were obtained via radial artery tonometry: aortic or central pulse pressure (C_PP_, the difference between convolved aortic systolic and diastolic pressure) and augmentation index (AIx, reflecting the timing and quantum of wave reflection from branch points or areas of impedance mismatch which are determined by aortic stiffness and arteriolar tone). In addition to PWV, a vascular health composite score (zComposite [28]) was computed as a cardiovascular health outcome:

zComposite = average of Z_PWV_+Z_CPP_+Z_AIx_.

### Statistical analysis

Group differences in demographic characteristics, time spent in each movement behaviour (PA, SB and sleep) and cardiometabolic outcomes were evaluated using analysis of variance (ANOVA) tests, and statistical significance was set at *p* < 0.05.

Compositional data analyses (CoDA) were conducted in the working-age and older adults to examine the relationship between cardiometabolic risk factors (BRI, BMI, PWV and zComposite), and the composition of PA, SB and sleep across an average day. This approach appropriately accounts for the co-dependent nature of time-use data by modelling log-ratios (log-transformed ratios between movement behaviours), thereby describing relative time spent in different behaviours rather than absolute time in any single behaviour. Specifically, we used isometric log-ratio (ilr) pivot coordinates to represent and adjust for the 24-hour movement behaviour composition [29]. In the current study, non-wear time was considered sedentary to preserve a 24-hour profile and avoid inflation of activity. Cardiometabolic outcomes were analysed in the combined working-age and older adult sample to maximise statistical power and provide more stable estimates for the compositional analyses.

Results from the compositional models are presented in three stages. First, overall CoDA regression results visualized using ternary plots, illustrate associations between cardiometabolic outcomes and the 24-hour composition of PA, SB, and sleep. Second, omnibus time substitution analyses are presented using forest plots, estimating associations when a fixed duration of time is reallocated to one behaviour proportionally from the others (e.g., 30 min/day to PA from SB and sleep). For example, reallocating 30 min/day to PA from a baseline composition of PA = 300 min, SB = 600 min, sleep = 540 min, for example, would involve proportionally reducing time from SB and sleep based on their relative contributions to the remaining 1140 min (600 + 540). In this example, approximately 16 min would be taken from SB and 14 min from sleep. Third, pairwise (isotemporal) substitution analyses were conducted for behaviour-pairs significant in the omnibus models, estimating associations when time is directly substituted from one behaviour to another (e.g., replacing SB with PA). All estimates are expressed relative to the sample’s mean 24-hour composition and represents the expected change in cardiometabolic outcomes associated with different patterns of time substitution for a hypothetical average individual. Substitution effects were obtained by using the compositional models to estimate outcome differences relative to this mean behaviour composition for specific substitutions of time (e.g. ± 30 min) between behaviours.

Age and sex were included as covariates in all CoDA models, as well as the sleep fragmentation index (SFI) to account for movement during sleep. Models were further adjusted for self-reported hypertension, diabetes, hyperlipidemia, and smoking status.

Analyses were conducted in R (version 4.4.2), with compositional data analyses performed using the epicoda package [30].

## Results

### Sample characteristics

After exclusion of ineligible records (see Fig. 1), the final analyses included 1,967 participants (500 university students [50.8% female], 437 working adults [61.3% female], and 1,030 older adults [53.5% female]) who contributed 48,535 valid days of movement behaviour data. Table 1 and Additional File 1: Supplementary Table 2 summarize demographic information and cardiometabolic health indicators.

**Table 1:** Sample characteristics

	University Students *N* = 500	Working Adults *N* = 437	Older Adults *N* = 1,030	F/χ²/t-values
Age (y)	19.9 (1.3)	41.1 (10.0)	74.0 (2.6)	20,258***
Female (%)	254 (50.8%)	268 (61.3%)	551 (53.5%)	11.39**
Wear time (min)	1386 (30)	1374 (34)	1377 (31)	20.55***
PA Duration (min)	320.28 (86.35)	346.55 (84.74)	420.28 (129.27)	160.8***
MVPA Duration (min)	45.78 (21.82)	55.03 (29.55)	80.26 (54.82)	123.4***
LPA Duration (min)	274.50 (75.11)	291.52 (71.96)	340.02 (102.95)	104.1***
Sedentary Time (min)	633.89 (77.34)	581.28 (78.92)	500.86 (108.77)	352.8***
Sleep (min)	431.75 (48.33)	445.77 (55.23)	455.62 (79.01)	21.27***
BMI (kg/m^2^)	21.72 (3.34)	24.69 (4.70)	23.99 (3.57)	81.53***
BRI	-	3.61 (1.32)	4.71 (1.38)	13.25***
Systolic BP (mmHg)	-	119.29 (14.23)	135.93 (18.30)	17.60***
Diastolic BP (mmHg)	-	75.80 (9.90)	76.37 (9.77)	0.97
PWV (m/s)	-	5.63 (1.39)	9.26 (2.07)	35.00***
CPP (mmHg)	-	43.52 (8.04)	53.95 (12.63)	16.67***
AIx	-	18.36 (13.62)	27.65 (9.24)	12.36***
Values represent mean (SD)				

*** *p* < 0.001; *p* < 0.05

PA – physical activity, MVPA – moderate-to-vigorous physical activity, LPA – light physical activity, BMI – body mass index, BRI – body roundness index, PWV – pulse wave velocity, BP – blood pressure, CPP – central pulse pressure, AIx – augmentation index

Working adults and older adults in the current analyses were generally healthy, with an average BMI of 24.25 kg/m^2^, BRI of 4.29 and PWV of 7.86 m/s. A small but significant age-related decrease was observed for BMI, r(1120) = -0.07, *p* < 0.05; whereas significant age-related increases were observed for BRI, r(1110) = 0.35, *p* < 0.001; PWV, r(1108) = 0.71, *p* < 0.001; and zComposite, r(1103) = 0.63, *p* < 0.001, consistent with existing literature on aging and cardiovascular risk markers [31].

### Age-related differences in 24 h movement behaviours

Greater PA and lower SB across the 24 h day was observed with older age (Table 1). PA was longest in the older adults (420.28 min), followed by the working adults (346.55 min) and university students (320.28 min); this trend was evident on both weekdays and weekends (Additional File 1: Supplementary Table 3) and when considering both light and moderate-to-vigorous physical activity (Table 1). Conversely, SB was the shortest in the older adults (500.86 min), followed by the working adults (581.28 min) and university students (633.89 min).

Time allocated for sleep across the 24 h day was also longest among the older adults (455.62 min vs. 445.77 min in the working adults and 431.75 min in the university students (Table 1). However, when considering main sleep metrics, actual time spent asleep was shortest in older adults (Additional File 1: Supplementary Table 1) due to more fragmented sleep - resulting in overall higher sleep fragmentation (SFI) scores, increased wake after sleep onset (WASO), and reduced sleep efficiency (Additional File 1: Supplementary Table 1).

**Fig. 2:**
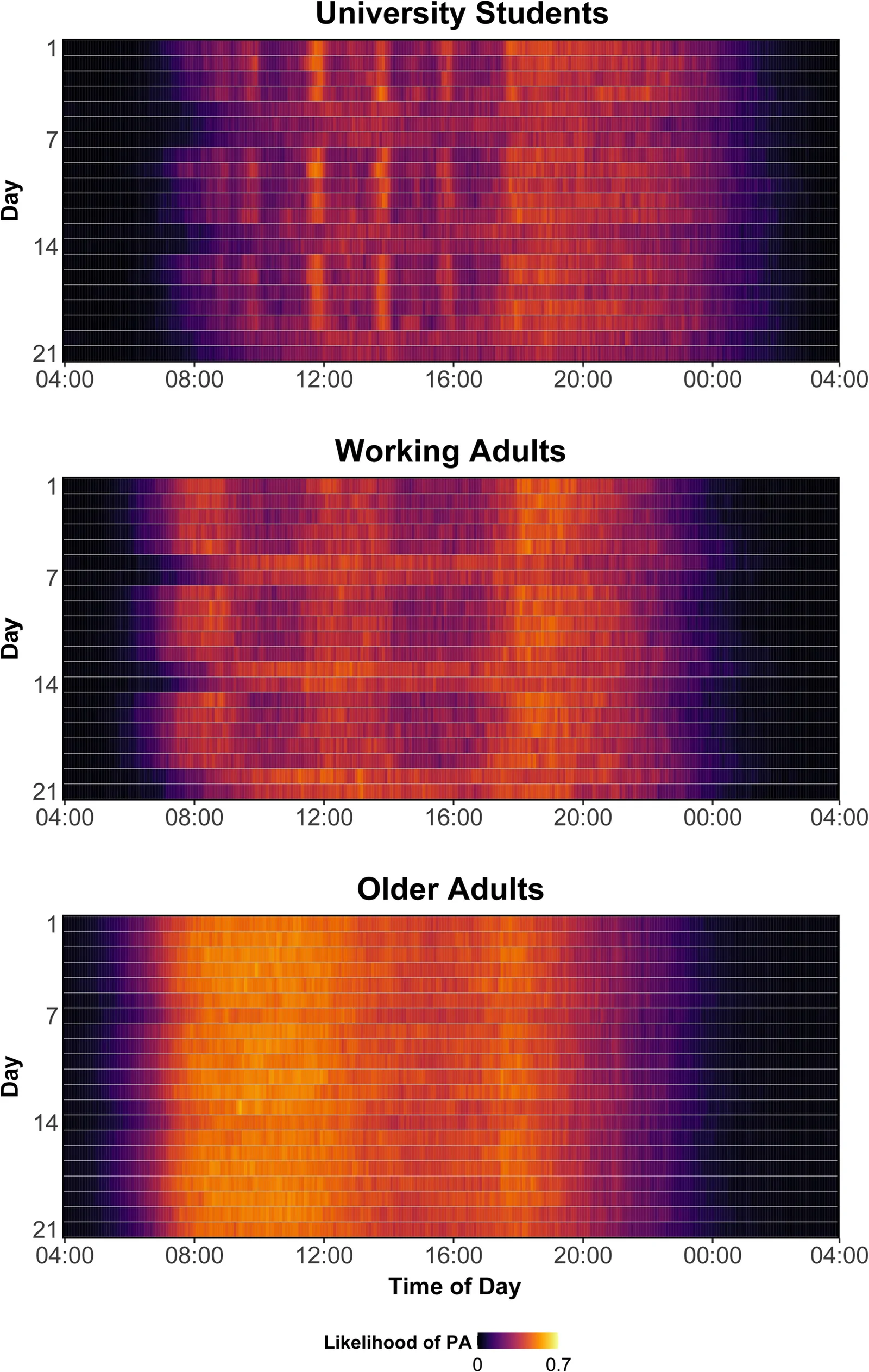
Heatmaps depicting likelihood of PA across three groups of adults (university students, working adults, older adults). Each row of a heatmap represents a 24 h day, while each heatmap has 21 rows (days) starting from the first Monday recorded for each participant’s four-week collection period. Each cell of a row corresponds to a 5-min epoch across the 24 h day. Heatmaps were averaged across participants within each group. Colours in each cell represent the likelihood of PA occurrence in that age group (cool – low, hot – high). Among the three age groups, older adults had a higher likelihood of PA throughout the daytime, while students and working adults tended to have greater PA around class/work hours on weekdays. Note: Day 5 in the student group (top panel) was a public holiday, hence its activity distribution was more similar to a weekend (Days 6–7) than a weekday (Days 1–4)

Heatmaps (Fig. 2) indicate that older adults were active throughout the day, except for a dip during the afternoon. In contrast, both students and working adults had lower likelihood of weekday PA, with activity concentrated at specific times. In students, weekday PA was most likely to occur around the start and end of lessons, reflecting movement from class to class. Working adults demonstrated higher PA likelihood during commute hours in the mornings and evenings, as well as around lunchtime.

On the weekends, PA likelihood was markedly lower and started later in university students compared to weekdays. Although PA likelihood in working adults appeared to be preserved on weekends, its start time was delayed and more evenly spread out over the day. These observations lie in contrast to older adults in whom the likelihood and timing of PA was similar across weekdays and weekends.

### Impact of time reallocation between movement behaviours on cardiometabolic measures by CoDA

**Fig. 3:**
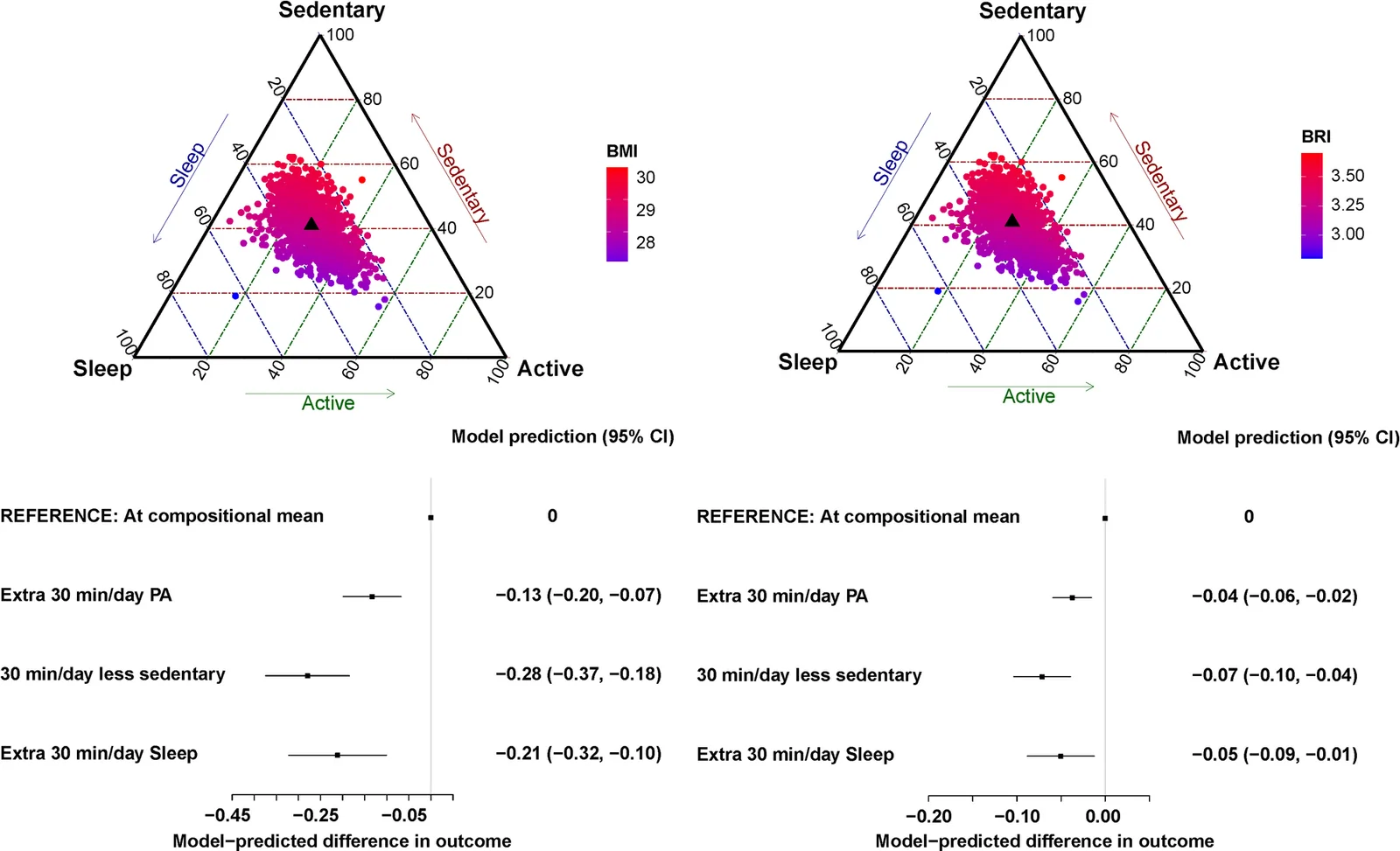
Compositional regression analyses showing that reallocating time to more physical activity (PA) was associated with lower BMI and body roundness index (BRI). Each dot in the ternary plots represents the 24 h activity composition of one participant, with the solid black triangle indicating the sample mean composition. The distribution of dots is identical across all ternary plots. Dot colours are weighted by the regression beta estimates, illustrating that participants with higher PA had lower BMI and BRI (blue shading toward the more active corner). The accompanying forest plots display substitution model results, demonstrating that reallocating 30 min of sedentary time to PA or sleep was associated with lower BMI and BRI.

**Fig. 4:**
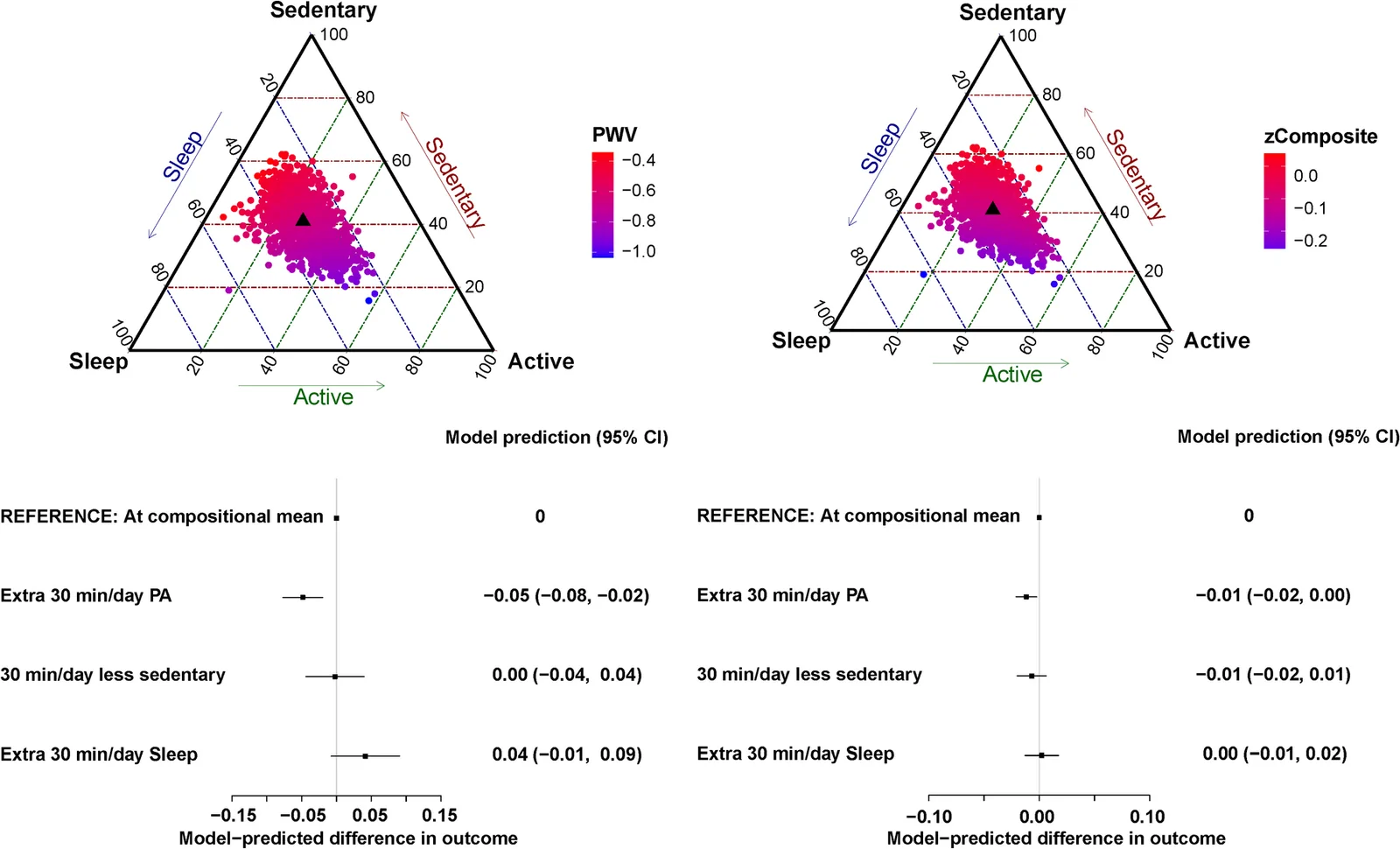
Compositional regression analyses showing that reallocating time to more physical activity (PA) was associated with lower cardiometabolic risk. Each dot in the ternary plots represents the 24 h activity composition of a single participant, with the solid black triangle indicating the sample mean composition. The distribution of dots is identical across all ternary plots. Dot colours are weighted by the regression beta estimates, illustrating that participants with higher PA had lower PWV and zComposite (blue shading toward the more active corner). The accompanying forest plots display substitution model results, demonstrating that reallocating 30 min more PA was associated with lower PWV and zComposite.

Overall CoDA regressions show that increasing PA, by taking time from other behaviours, is associated with lower BMI (beta=-1.16, 95% CI=-1.89 to -0.73), BRI (beta=-0.35, 95% CI=-0.6 to -0.1), PWV (beta=-0.57, 95% CI=-0.89 to -0.24) and zComposite (beta=-0.12, 95% CI=-0.22 to -0.02) (ternary plots; Figs. 3 and 4).

To facilitate interpretation of these effect sizes, omnibus substitution analyses (forest plots; Figs. 3 and 4) show that adding 30 min of PA proportionally from other behaviours was related to decreases in BMI (-0.13 kg/m^2^), BRI (-0.04), PWV (-0.05 m/s) and zComposite (-0.01). Similarly, adding 30 min of sleep from other behaviours was associated with lower BMI (-0.21 kg/m^2^) and BRI (-0.05), while reducing 30 min of SB related to lower BMI (-0.28 kg/m^2^) and BRI (-0.07).

Isotemporal (pairwise) substitution analyses between PA and SB further show that increasing PA time by taking time from SB related to lower BMI, BRI, PWV and zComposite (Additional File 1: Supplementary Fig. 2). Increasing sleep, also by taking time from SB, was related to lower BMI and BRI (Additional File 1: Supplementary Fig. 3). Time reallocations between PA and sleep were not significantly associated with cardiometabolic outcomes.

## Discussion

Using objective wearable data across three adult cohorts in Singapore, we showed that contemporary patterns of physical activity and sedentary behaviour no longer conform to long-standing epidemiological assumptions in which older adults are the least active and working-age or older adults the most sedentary [5–8]. Notably, we observed that university students in our sample averaged only 320.28 min of daily physical activity compared to 420.28 min in the older adults, and they also spent 633.89 min being sedentary, compared to 500.86 min in the older adults.

While lower PA levels in students can be expected on weekdays due to classes and other academic commitments, PA declined by a further ~ 30-min on the weekends – which could suggest that opportunities to increase PA were not taken. While sedentary time also decreased on the weekends, this reduction was offset by increased sleep time, consistent with weekend “catch-up” sleep [32]. Among working-age adults however, PA duration was largely preserved on the weekends. The onset of PA shifted later and was distributed more evenly across the day. Reductions in sedentary time during the weekends were also primarily replaced by longer sleep. Remarkably, older adults exhibited the highest total PA on both weekdays and weekends, averaging ~ 60–100 min more per day than the students and working-age adults. Time spent sedentary was also the lowest; averaging ~ 15–140 min less per day. Older adults allocated the longest time for sleep (time in bed) on average, but actual total sleep time was the shortest - consistent with established age-related declines in sleep efficiency and increased sleep fragmentation [33]. They also showed the greatest stability in PA, SB, and sleep across the week. Taken together, these findings indicate that older adults’ movement behaviours reached favourable levels and were well distributed temporally across the week, despite their shorter and more fragmented sleep. Conversely, students, despite a greater capacity to be more physically active, did not appear to fully capitalise on this even on weekends with fewer competing commitments.

In the working-age and older adult groups, unfavourable movement profiles were associated with adverse cardiometabolic profiles. The use of compositional methods which integrate physical activity, sedentary time, and sleep as co-dependent components of a finite 24 h day allowed us to quantify how trade-offs between movement behaviours relate to cardiometabolic health [29]. In the omnibus analyses, reallocating 30 min/day toward physical activity from the remaining movement behaviours was associated with lower adiposity (BMI and BRI) as well as more favourable vascular health indicators (PWV and zComposite). In contrast, reallocating time toward sleep was associated primarily with lower adiposity, while reducing sedentary behaviour was similarly linked to lower adiposity and improved overall vascular health. Pairwise substitution analyses further indicated that these associations were largely driven by the exchange of sedentary behaviour with either physical activity or sleep. Specifically, replacing sedentary time with physical activity was associated with favourable differences across both adiposity and vascular outcomes, whereas replacing sedentary time with sleep was associated with lower adiposity alone.

Collectively, these findings suggest that greater physical activity is associated with the broadest cardiometabolic benefits, while associations with longer sleep are largely confined to adiposity-related measures. The absence of significant associations when time was exchanged between physical activity and sleep further suggests that both behaviours may be beneficial relative to sedentary behaviour, rather than one being consistently superior to the other. Consequently, reducing sedentary time—whether through greater physical activity or longer sleep duration—may be more important than shifting time between physical activity and sleep itself. These findings also align with prior studies [34–36] reporting cardiometabolic benefits when sedentary time is substituted with PA or sleep (particularly for short sleepers [37]) - with improvements observed across adiposity, lipid profiles, and glycemic indices.

Our findings of greater inactivity among university students contrast with earlier Singaporean surveys [38], past actigraphic studies [39], and Western data showing age-related declines in physical activity [40]. While underlying drivers of increased sedentary behaviours and decreased physical activity were not directly assessed, they likely reflect broader societal shifts associated with pervasive digitalisation of education, work, transport, and leisure. These habits – particularly pronounced amongst the young who have grown up with this way of life, have been facilitated by the widespread availability and affordability of portable electronic devices, streaming media, online education, food delivery, and ride-hailing services, all of which have collectively reduced the need even for incidental movement [41, 42]. Although cardiometabolic outcomes were not assessed in the university student cohort, their relatively low physical activity and high sedentary time may represent an unfavourable movement behaviour profile that warrants prospective follow-up to determine whether it translates into increased cardiometabolic risk later in adulthood. Globally, physical inactivity is already estimated to cost health care systems USD 53.8 billion annually, while in Singapore, the economic cost of physical inactivity is estimated to be USD 201 million [43, 44]. Modelling studies further suggest that a 10y population-wide physical activity programme in Singapore could lead to a reduction in health care costs of SGD (Singapore dollar) 448 million through the prevention of diabetes, hypertension, and related complications [43], underscoring the substantial health and economic benefits of scalable, population-level interventions that increase physical activity and reduce sedentary behaviour. Preventive strategies must therefore happen now, not only by encouraging promotion of weekday bouts of activity but also by considering how urban environments and digital environments can be redesigned to restore incidental and habitual movement in younger generations.

This study has several notable strengths, including the use of objective, high-resolution wearable data collected under free-living conditions and harmonised across three cohorts spanning early adulthood to older age. The integration of physical activity, sedentary behaviour, and sleep within a 24-h compositional framework further allowed us to examine realistic behavioural trade-offs as they occur in daily life. Against this backdrop, several limitations warrant consideration. First, the findings are derived from convenience samples of individuals who volunteered for a consumer-technology tracking study. Although devices were provided to ensure that this would not be a barrier to participation, such individuals may not be representative of the general population. Second, confounding by sociodemographic factors apart from age cannot be fully excluded. The older adult cohort was restricted to ethnic Chinese participants by design, although this also comprised the majority ethnic group in the other two cohorts. However, in a separate sensitivity analysis limited to only participants of Chinese ethnicity with equal male-female representation across all cohorts, we observed the same age-related gradients in movement behaviour profiles as in the full cohort, suggesting that the findings were not driven by differences in ethnicity or sex composition (Additional File: Supplementary Table 4). Nonetheless, we could not completely account for socioeconomic status through housing information, as over half of the university students resided on campus. Third, although 5-minute MET intervals were used here, the optimal time scale for capturing health-relevant movement patterns remains uncertain, given the limited experimental evidence comparing short and long activity bouts. Finally, substitution estimates should be interpreted as hypothetical associations. Further experimental and intervention studies are needed to determine the feasibility and real-world implications of these reallocations and to assess whether the estimated changes in behaviour translate into meaningful health benefits.

## Conclusions

Using a compositional framework, we found that university students accumulated less physical activity and more sedentary behaviour than both working-age and older adults, highlighting young adulthood as a potentially important target for movement behaviour interventions. Furthermore, in the working-age and older adults who had cardiometabolic assessments, reallocating time away from sedentary behaviour was associated with more favourable adiposity and vascular health profiles. Together, these findings underscore the importance of considering the entire 24-hour movement behaviour composition, rather than individual behaviours in isolation, when evaluating cardiometabolic health and informing public health strategies.

## Supplementary Information

Below is the link to the electronic supplementary material.

Supplementary Material 1

## Data Availability

The data in this article are available upon reasonable request with a signed data access agreement. For further information, please contact the corresponding author.

## Footnotes

1
https://support.ouraring.com/hc/en-us/articles/360025576833-How-Oura-Measures-Steps-Activity.

## Abbreviations

AIx: augmentation index
ANOVA: analysis of variance
BMI: body mass index
BP: blood pressure
BRI: body roundness index
CoDA: compositional data analyses
CPP: central pulse pressure
LPA: light physical activity
MET: metabolic equivalent
MVPA: moderate-to-vigorous physical activity
PA: physical activity
PWV: pulse wave velocity
SB: sedentary behaviour
SD: standard deviation
SGD: Singapore dollar
USD: US dollar
