# Supplementary material for: 24h movement behaviours in university students compared to working age and older adults: wearable-based evidence and cardiometabolic health implications
Source: BMC Med. 2026 Aug 29;24:485. doi: 10.1186/s12916-026-05192-1 (PMC13573439; doi:10.1186/s12916-026-05192-1)
Supplement: Supplementary file 1 [file 12916_2026_5192_MOESM1_ESM.docx]

**Supplementary Methods**

(A) **Inclusion and exclusion criteria**

Students were eligible for inclusion if they were first-year full-time undergraduates enrolled at the National University of Singapore (NUS in the 2023-2024 academic year, and if they were aged between 18 and 25. Working adults were eligible if they were full-time staff members at NUS, aged between 21 and 70, whose work did not involve shift work, who were not pregnant or breast-feeding, and who reported no diagnosed sleep, neurological or psychological disorders. Older adults were recruited from the SG70 project, which is nested in the Singapore Chinese Health Study (SCHS). The SG70 study, which started in September 2021, enrolled 1200 surviving participants aged 65–80 from the original SCHS cohort to comprehensively assess ageing outcomes and its associated factors.

(B) **Study period**

The Oura ring captured continuous movement behaviour data throughout each of the following study periods: (1) University students – across five months of their first semester at university (August–December 2023), (2) Working adults - across one year of full-time employment (rolling recruitment from October 2024 to January 2025), and (3) Older adults - across four weeks (rolling recruitment from September 2021 to August 2024). To facilitate comparability across cohorts, analyses in the present study were standardised to four-week data segments. For the student sample, data from Weeks 3 to 6 of the semester were used to allow for acclimatisation to university routines; for the working and older adult sample, data were drawn from the earliest four-week period following enrolment.

(C) **Oura data pipeline**

Full details are described in a separate study.^1^ For analysis of sleep metrics from the Oura Ring (Supplementary Table 2), only main sleep periods, i.e., those with the longest TST between 1800h the previous day to 1800h the present day were considered. Days with sleep periods of ≥15h were excluded as these likely represented inaccurate, or unrepresentative sleep episodes (e.g., due to illness). If a day’s longest recorded TST was <3h, or if no sleep was recorded, only days with minimum wear time of at least 20h were included to ensure that these were not missed due to non-wear or other technical reasons.

(D) **Omnibus substitution between MVPA, LPA, Sedentary behaviour and Sleep**

Substituting time to MVPA by proportionally taking time off LPA, sedentary behaviour and sleep was related to lower BMI and BRI, but not with PWV and zComposite. Substituting time to LPA by proportionally taking time off MVPA, sedentary behaviour and sleep was related to lower BMI, PWV and zComposite (Supplementary Figure 1).

The lack of significant associations when substituting time to MVPA for PWV and zComposite likely reflects characteristics of the current sample rather than an absence of true physiological benefit. Participants—both working adults and older adults—already accumulated high levels of MVPA on a daily basis (≈55 min/day and ≈80 min/day, respectively). Such levels exceed typical population averages and approach thresholds at which additional MVPA may yield diminishing marginal returns.

In this context, the substitution models faced a ceiling effect: when MVPA is already high and shows limited variability, adding an additional 30 minutes may not meaningfully shift cardiometabolic outcomes. In contrast, reallocating time to LPA showed significant associations with PWV and zComposite, likely because LPA had a broader distribution and more room for measurable behavioural change within this sample.

Thus, the non-significant MVPA findings likely stem from behavioural saturation and restricted variability, which attenuate detectable associations in compositional models.

**(E) Isotemporal substitution analyses**

Isotemporal (pairwise) substitution analyses showed that reallocating time from sedentary behaviours to PA was related to decreased BMI, BRI, PWV and zComposite (Supplementary Figure 2). Reallocating time from sedentary behaviours to sleep was related to lower BMI and BRI (Supplementary Figure 3).

**Supplementary Table 1. Oura-derived sleep metrics (main sleep period)**

|  | University Students  N=500 | Working  Adults  N=437 | Older  Adults  N=1,030 | F-value |
| --- | --- | --- | --- | --- |
| TIB (min) | 440·85 (38·64) | 443·89 (47·08) | 454·15 (58·88) | 13·28*** |
| TST (min) | 394·63 (34·48) | 383·95 (43·01) | 365·83 (53·96) | 68·12*** |
| WASO (min) | 21·96 (13·22) | 30·85 (16·92) | 60·79 (29·27) | 546·5*** |
| Efficiency (%) | 89·65 (2·70) | 86·69 (3·71) | 80·80 (6·30) | 573·1*** |
| SFI | 31·15 (7·95) | 32·21 (8·30) | 41·74 (11·44) | 251·6*** |
| Values represent mean (SD) | | | |  |

*** p<0.001

TIB: time-in-bed (time spent in bed)

TST: total sleep time (time spent asleep out of the time spent in bed) WASO: wake after sleep onset

Efficiency: 100*TIB/TST

SFI: sleep fragmentation index

**Supplementary Table 2. Additional sample characteristics**

|  | University Students  N=500 | Working  Adults  N=437 | Older  Adults  N=1030 | F/χ²/t-values |
| --- | --- | --- | --- | --- |
| **Ethnicity** |  |  |  |  |
| Chinese | 435 (87·00%) | 341 (78·03%) | 1030 (100·00%) | 219·73*** |
| Malay | 16 (3·20%) | 24 (5·49%) | - |  |
| Indian | 31 (6·20%) | 39 (8·92%) | - |  |
| Others | 18 (3·60%) | 33 (7·55%) | - |  |
| **Primary Residence** |  |  |  |  |
| HDB* 1-3 room | 22 (11·40%) | 68 (15·60%) | 233 (22·62%) | 265·72*** |
| HDB* 4-5 room | 116 (60·10%) | 206 (47·25%) | 746 (72·43%) |  |
| Private Condominium/ Apartment | 34 (17·62%) | 124 (28·44 %) | 39 (3·79%) |  |
| Landed Property^‡^ | 21 (10·88%) | 31 (7·11%) | 12 (1·17%) |  |
| On Campus | 265 | - | - |  |
| Off Campus Shared Housing | 39 | - | - |  |
| NA | 3 | 8 | - |  |
| **Smoking Status** |  |  |  |  |
| Non-smoker | - | 397 (90·85%) | 835 (81·07%) | 28·77*** |
| Ex-smoker | - | 22 (5·03%) | 154 (14·95%) |  |
| Current smoker | - | 16 (3·66%) | 41 (3·98%) |  |
| NA | - | 2 | - |  |
| **Disease** | - |  |  |  |
| Angina | - | 1 (0·23%) | 48 (4·66%) | 203·32*** |
| Arthritis | - | 8 (1·84%) | 369 (35·83%) |  |
| Asthma |  | 29 (6·67%) | 48 (4·66%) |  |
| Cancer | - | 8 (1·84%) | 112 (10·87%) |  |
| Chronic lung disease | - | 1 (0·23%) | 7 (0·68%) |  |
| Coronary artery bypass graft or angioplasty | - | 1 (0·23%) | 81 (7·86%) |  |
| Diabetes | - | 9 (2·07%) | 272 (26·41%) |  |
| Gout | - | 6 (1·38%) | 86 (8·35%) |  |
| Heart attack | - | 1 (0·23%) | 100 (9·71%) |  |
| Heart failure | - | 3 (0·69%) | 12 (1·17%) |  |
| Hip fracture |  | 2 (0·46%) | 6 (0·58%) |  |
| Hypercholesterolaemia/Hyperlipidaemia | - | 30 (6·90%) | 726 (70·49%) |  |
| Hypertension (high blood pressure) | - | 35 (8·05%) | 635 (61·65%) |  |
| Kidney disease | - | 2 (0·46%) | 21 (2·04%) |  |
| Other bone fracture | - | 12 (2·76%) | 200 (19·42%) |  |
| Stroke | - | 1 (0·23%) | 45 (4·37%) |  |
| NA | - | 2 | - |  |
| Values represent mean (SD) | | | |  |

* HDB: government-supported public housing in the form of high-rise flats, developed and managed by the Housing and Development Board.

^‡^ Landed property: terraced, semi-detached, or detached house.

Note: Statistical analyses were conducted across fields comparable across all three cohorts, and excluded non-responses (NA).

**Supplementary Table 3. 24h movement behaviours by weekday/weekend**

|  | University  Students  N=500 | Working  Adults  N=437 | Older  Adults  N=1,030 | F-value |
| --- | --- | --- | --- | --- |
| Weekday PA Duration (min) | 329·72 (90·67) | 344·47 (85·07) | 424·69 (133·23) | 1883*** |
| Weekend PA Duration (min) | 299·31 (100·02) | 352·02 (104·62) | 409·23 (129·87) | 757·4*** |
| Weekday Sedentary Time  (min) | 642·05 (78·71) | 602·93 (82·56) | 498·57 (111·41) | 4268*** |
| Weekend  Sedentary Time (min) | 615·56 (93·06) | 522·23 (94·43) | 506·62 (111·95) | 884·3*** |
| Weekday Sleep (min) | 414·65 (51·71) | 426·67 (54·94) | 453·27 (81·95) | 430·4*** |
| Weekend Sleep  (min) | 469·68 (63·80) | 497·98 (74·40) | 461·36 (82·51) | 126·0*** |
| Values represent mean (SD) | | | |  |

*** p<0·001

PA: physical activity (light + moderate-to-vigorous)

**Supplementary Table 4.** Sample characteristics of participants of Chinese ethnicity, with equal representation of males and females. As females were overrepresented across all cohorts, the most sedentary females were excluded from each cohort to match the number of male participants, yielding a more conservative sensitivity analysis. Consistent with the findings from the full cohort, the same age-related gradient in movement behaviour profiles was observed.

|  | University Students  N=408 | Working  Adults  N=244 | Older  Adults  N=958 | F/χ²/t-values |
| --- | --- | --- | --- | --- |
| Female (%) | 204 (50.0%) | 122 (50.0%) | 479 (50.0%) | - |
| PA Duration (min) | 327.0 (85.6) | 368.5 (84.3) | 428.8 (128.7) | 122.9*** |
| MVPA Duration (min) | 46.7 (21.8) | 57.9 (29.3) | 82.8 (55.6) | 98.8*** |
| LPA Duration (min) | 280.3 (74.5) | 310.7 (72.0) | 346.0 (103.2) | 75.4*** |
| Sedentary Time (min) | 626.5 (74.4) | 558.6 (80.7) | 489.8 (103.7) | 313.2*** |
| Sleep (min) | 431.2 (48.9) | 445.3 (56.0) | 457.8 (79.7) | 21.2*** |
| Values represent mean (SD) | | | |  |

*** p<0.001; p<0.05

**Supplementary Figure 1.** Compositional regression analyses showing that reallocating time to more physical activity (MVPA or PA) was associated with lower zComposite, BMI and body roundness index (BRI).

**Supplementary Figure 2.** Isotemporal (pairwise) substitution results between physical activity (PA) and sedentary behaviours on PWV, zComposite, BMI, and BRI.

**Supplementary Figure 3.** Isotemporal (pairwise) substitution results between rest/sleep and sedentary behaviours on PWV, zComposite, BMI, and BRI.
